# Hand-Camera Coordination Varies over Time in Users of the Argus^®^ II Retinal Prosthesis System

**DOI:** 10.3389/fnsys.2016.00041

**Published:** 2016-05-06

**Authors:** Michael P. Barry, Gislin Dagnelie

**Affiliations:** ^1^Department of Biomedical Engineering, Lions Vision Research Center, Johns Hopkins UniversityBaltimore, MD, USA; ^2^Department of Ophthalmology, Lions Vision Research Center, Johns Hopkins UniversityBaltimore, MD, USA

**Keywords:** retinal implant, visual prosthesis, Argus II, hand-eye coordination, perceptual misalignment

## Abstract

**Introduction**: Most visual neuroprostheses use an external camera for image acquisition. This adds two complications to phosphene perception: (1) stimulation locus will not change with eye movements; and (2) external cameras can be aimed in directions different from the user’s intended direction of gaze. Little is known about the stability of where users perceive light sources to be or whether they will adapt to changes in camera orientation.

**Methods**: Three end-stage retinitis pigmentosa patients implanted with the Argus II participated in this study. This prosthesis stimulated the retina based on an 18° × 11° area selected within the camera’s 66° × 49° field of view. The center of the electrode array’s field of view mapped within the camera’s field of view is the camera alignment position (CAP). Proper camera alignments minimize errors in localizing visual percepts in space. Subjects touched single white squares in random locations on a darkened touchscreen 40 or more times. To study adaptation, subjects were given intentional CAP misalignments of 15–40° for 5–6 months. Subjects performed this test with auditory feedback during (bi-)weekly lab sessions. Misaligned CAPs were maintained for another 5–6 months without auditory feedback. Touch alignment was tracked to detect any adaptation. To estimate localization stability, data for when CAPs were set to minimize errors were tracked. The same localization test as above was used. Localization errors were tracked every 1–2 weeks for up to 40 months.

**Results**: Two of three subjects used auditory feedback to improve accuracy with misaligned CAPs at an average rate of 0.02°/day (*p* < 0.05, bootstrap analysis of linear regression). The rates observed here were ~4000 times slower than those seen in normally-sighted subjects adapting to prism glasses. Removal of auditory feedback precipitated error increases for all subjects. Optimal CAPs varied significantly across test sessions (*p* < 10^−4^, bootstrap multivariate analysis of variance (MANOVA)), up to 21–29° within subjects over the observed period. Across subjects, optimal CAPs showed an average rate of change of 0.39°/day (SD 0.36°/day).

**Conclusions**: Optimal CAPs varied dramatically over time for all subjects. Subjects displayed no adaptation to misaligned CAPs without feedback. Regular recalibration of CAPs may be required to maintain hand-camera coordination.

## Introduction

Current visual prosthesis designs intend to restore some hand-eye coordination to those who are otherwise blind, along with other domains of functional vision: visual information gathering (Geruschat et al., 2016), visually guided mobility (Ho et al., 2015), and reading/shape recognition (da Cruz et al., 2013; Stingl et al., 2013b). Efficacy tests for the Argus II (Ahuja et al., 2011), Alpha IMS (Stingl et al., 2013a), and Pixium Vision’s IRIS^®^^1^ have included pointing and reaching tasks. Devices that use intraocular photodiodes, such as the Alpha IMS and Pixium Vision’s PRIMA (Lorach et al., 2016), avoid potential localization problems with eye-camera misalignments by effectively placing the camera that controls stimulation inside the eye. The Argus II, IRIS (Luo and da Cruz, 2014), and other retinal prostheses, such as Bionic Vision Australia’s prototype suprachoroidal prosthesis (Ayton et al., 2014), however, require input from a camera outside of the eye. All future optic nerve, thalamic, and cortical prostheses will also require light to be captured away from the stimulating electrodes, and image capture will likely not be implemented inside the eye. With any of these devices for which the camera will be outside the eye, there exists the potential for eye movements to create misalignments between prosthesis users’ egocentric percept locations and the true locations of the corresponding stimuli.

Sabbah et al. (2014) demonstrated that misalignments between eye and camera orientation can generate predictable localization errors. When prosthesis users intentionally deviated their eyes while pointing at a light source, the locations to which they pointed were consistently deviated from the light source in the direction of their gaze. Such deviated perceptions are logical, as eye orientation is critical to how the brain integrates visual information into the perception of egocentric space. The stimulating electrodes were fixed to one location on the retina, and the interpretation of signals from that patch of retina changed with each eye movement, just as with native visual stimulation.

Considering the limitations of current prosthesis technology (Eiber et al., 2013), one might assume that hand-camera coordination errors caused by transient eye movements may be acceptable. Such errors might be assumed to be symmetrically distributed over time, so users could still have reasonable average accuracy. Even if that were the case, however, little is known about the long-term dynamics of visual percept localization in blind individuals. We do not know whether such localization is consistent over time, disregarding brief eye movements.

New prosthesis users, if their system uses an external camera, typically receive some form of camera alignment as part of their initial fitting or programming. Without such alignment, the camera will most likely be aimed in a direction different from that which the user will perceive as the source of light. Perceived percept locations are strongly bound to the electrode array’s position on the retina. The array’s field of view, based on input from the camera, must then be moved as close as possible to the perceived percept location to optimize localization accuracy. Camera alignment can be accomplished by physically moving the camera and/or changing what part of its field of view is sampled to drive stimulation. These modifications should ultimately minimize any apparent localization errors. If this is not performed, however, or not performed sufficiently well, it is unknown whether users will adapt to misalignments on their own.

Individuals with normal vision can quickly adapt to shifts in their vision, or even complete inversions of their visual fields, to correct localization and coordination errors introduced by wearing prism glasses. There is a possible reason, though, why one may not expect such adaptation from those using current visual prostheses. Held and Hein (1958) demonstrated that adaptation to perturbations in vision requires reafference, or sensory feedback corresponding to motor commands. Current prosthesis users can find it difficult to, e.g., distinguish light reflecting off their own bodies from that of other sources. Luo et al. (2015) attempted to address this problem by placing a flashing LED on an Argus II user’s finger, but found that it offered no benefit in localization performance. These results may imply that prosthetic vision is not yet able to provide meaningful reafference that would allow adaptation to camera misalignments.

The camera alignment process for users of prostheses with external cameras, both current retinal and future intracranial designs, cannot be optimized without knowing whether percept localization is generally stable and whether users can adapt to misalignments. If localization is stable and users can adapt to misalignments, then no external alignment may be necessary at all. Conversely, if localization is not stable and users cannot adapt to misalignments, regular camera alignments will be required for optimal hand-camera coordination. This study examines the localization behavior of Argus II users to help guide future prosthesis designs and programming strategies.

## Materials and Methods

### Subjects and Prosthesis Configuration

Three Argus II users (S1, S2, and S3) involved in the Argus II Feasibility Study (ClinicalTrials.gov: NCT00407602) participated in this research. One male and one female were implanted in June 2007, and one male was implanted in June 2009. All subjects suffered from end-stage retinitis pigmentosa and had the prosthesis implanted in the right eye. This research was approved by the Johns Hopkins Institutional Review Board and adhered to the tenets of the Declaration of Helsinki. Subjects all provided their informed consent to participate before research began.

Subjects’ camera alignment positions (CAPs) were controlled through computer software. The electrode array for each subject covered approximately 17.9° × 10.8° of visual field. The image captured by the prosthesis camera spanned 66° × 49° or 49° × 38°, depending on external hardware models. The array’s field of view could be chosen as any approximate 18° × 11° area from the camera’s wider field of view. The subsection of the camera’s field of view mapped as the array’s field of view served as the source information for stimulation. The point mapped as the center of the array’s field of view was designated as the CAP. Once configured, the CAP would remain as a fixed parameter in the subject’s video processing unit, but could be changed by connecting the system to a computer with specialized software. The minimum step size for selecting CAP coordinates in horizontal or vertical dimensions was 0.27°. This setup did not allow for tilting of the camera with respect to glasses frame. Although the system permits rescaling of the array’s field of view (Sahel et al., 2013), such that a smaller image can fill the array or a larger image can be shrunk to fit within the array, no rescaling was used in these experiments.

### Data Collection

Localization accuracy was primarily measured by asking subjects to touch solitary white square or circular targets that appeared on the black background of a touchscreen. The touchscreen covered an area of 37.5 cm × 30 cm and was positioned 36–38 cm away from the subject, depending on what was most comfortable for each subject. Head motion was not restricted, but subjects were encouraged to not move significantly closer or farther from the screen while they scanned for targets. Subjects were permitted to use both hands for touching the screen. Targets typically had 3 cm diameters, spanning about 4° of visual field, and appeared at random locations on the screen. Subjects completed up to 400 trials, broken into runs of 10–80 trials, as frequently as once per week for 3.5 years.

CAPs were intentionally misaligned from perceived percept locations to test whether subjects could adapt to such misalignments. Misalignments ranged 15°–40° away from optimal, and were maintained for approximately 1 year. Subjects were told that objects might appear to be in locations different from their true locations, and asked to report any detected misalignments. Any misalignments that were considered problematic by the subjects during daily use of the Argus II system were to be removed upon their report.

For the first 5–6 months of using misaligned CAPs, subjects’ localization accuracy and precision were tested as frequently as once per week. Localization tests during this period included auditory feedback, which informed the subject whether each response was correct, and if not, where the target was relative to the touched location on the screen. This feedback only specified direction, and did not specify distance. For example, if a subject touched anywhere below a target, the program’s feedback would be “It was higher.” Subjects did not have any opportunity to repeat trials, and the next target automatically appeared in a random location after the program finished providing feedback. If any improvement in accuracy was observed by the end of this period, CAPs were maintained and auditory feedback during testing was removed. Localization data were collected for another 5–6 months to determine whether any previously observed trend could continue without in-lab auditory feedback.

Prior to and following the period of localization tests with misaligned CAPs, subject CAPs were optimized each week during psychophysics testing sessions, or as frequently as subjects could be seen. Specifically, localization errors were used to calculate appropriate CAP adjustments to minimize errors, and subjects repeated testing with the new CAP. Localization and estimated optimal CAP data were collected over a total period of 3.5 years. Whenever subjects’ average responses were noticeably inaccurate, CAPs were adjusted and localization tests were repeated until average error was less than 1° or CAPs could not be further adjusted in the necessary directions.

When attempting to optimize a subject’s CAP, the number of percepts seeming to appear outside of the screen boundaries was minimized by implementing a margin near the screen’s border in which no targets could appear. Without such margins, any targets that appeared next to the screen border could have restricted the measurement of localization errors and, more importantly, provided unintended tactile feedback to the subject. Typical margins ranged from 4 to 23 cm, depending on each subject’s typical magnitude and direction of errors. Asymmetric margins were used when subjects had large localization errors in one direction, and the limits of the camera’s visual field would not allow for an eccentric enough CAP to correct the errors.

### Data Analysis

To determine whether subjects could adapt to misaligned percepts, localization errors, in degrees of visual field, were averaged for each trial run. Data analysis focused on the distance of each resulting error centroid from the origin, such that a centroid distance of 0° would imply perfect accuracy.

Linear regression, using the ordinary least squares method, of centroid distances vs. time was employed to identify any trends in localization accuracy. The significance of any effect of time was determined by bootstrap resampling of data pairs; see Henderson (2005) for an overview of bootstrap analysis. 10^4^ bootstrap sample distributions were generated for each analysis, and regression lines were computed for each bootstrap distribution. Bias-corrected and accelerated (BC_a_) 95% confidence intervals were calculated for each empirical regression line’s slope and ordinate values across the relevant domain. Empirical influence values were determined using the ordinary jackknife method. Any slope significantly less than 0, combined with a change in average centroid distance no less than 2°, half the width of typical targets, was considered an indication of adaptation.

When trying to minimize localization errors and determine localization stability, pooled errors for each testing day were used to estimate optimal CAPs for each subject. Estimated optimal CAPs were calculated for each test trial, and CAP estimates for all trials in a day were averaged to estimate an optimal CAP to minimize errors for that day. Trial CAP estimates for each subject, grouped by day, were also analyzed directly through a one-way parametric bootstrap multivariate analysis of variance (MANOVA) using a multivariate Wald-type test statistic (Konietschke et al., 2015); 10^4^ bootstrap distributions were generated for each test. This MANOVA variant has particular advantages here as it does not require homogeneous covariance matrices or equal group sizes to estimate probability distributions. Standard bivariate normal distributions were used for determining confidence ellipses around optimal CAP estimates in plots.

The horizontal components of estimated optimal CAPs for subject S3 were additionally plotted against time to demonstrate the consistency of shifts in this dimension. The regression line for this plot and its confidence bands were calculated using the same methods as described above.

Data corresponding to intentional misalignments, as while adaptation was being tested, were excluded from analyses of localization stability. Further, trial runs with CAPs that were more than 5° away from optimal settings and not limited by the camera’s visual field boundaries were also excluded from analyses.

## Results

**Figure 1:**
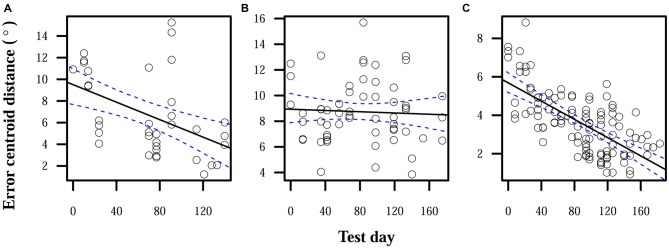
**Reduction in localization error when auditory feedback was present.** Data points each represent the error centroid distance from the origin for one trial run. Regression lines are shown for each distribution of centroid distances over time. 95% confidence bands are shown with dashed lines. **(A)** S1: slope = −0.04°/day, intercept = 9.5°. **(B)** S2: slope = −0.002°/day, intercept = 8.9°. **(C)** S3: slope = −0.02°/day, intercept = 5.7°.

During the period in which subjects used misaligned CAPs and testing included auditory feedback, two of three subjects showed some significant improvement in accuracy. Improvement was very slow, averaging 0.02°/day. Subject S1 showed a total average decrease in error centroid distance of 6° during this period. S2’s decrease in centroid distance was not statistically significant, and only fell on average by 0.4°. S3 showed a significant decrease of 4°. Figure 1 shows the reduction in error centroid distances over time.

**Figure 2:**
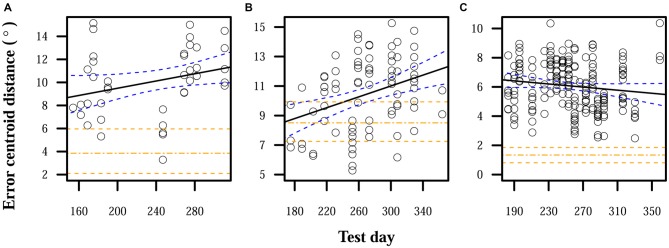
**Localization errors when auditory feedback was not present.** Data points each represent the error centroid distance from the origin for one trial run. Regression lines are shown for each distribution of centroid distances over time. The values of the regression lines at their last time points in corresponding plots in Figure 1 are shown as horizontal dot-dashed lines. 95% confidence bands are shown with dashed lines, for both the given regression lines and the final regression line values from Figure 1. **(A)** S1: slope = 0.02°/day, intercept = 8.7°. **(B)** S2: slope = 0.02°/day, intercept = 8.7°. **(C)** S3: slope = −0.005°/day, intercept = 6.5°.

When auditory feedback was removed, localization errors significantly increased over time for S1 and S2. S3 displayed a nonsignificant reduction in errors over time, but the expected error centroid distance for the last time point of the linear model of the feedback-ON period and its confidence interval were lower than any observed distance in the feedback-OFF period. Final error magnitudes were thus higher at the end of this observation period than before auditory feedback was removed. Comparing linear model expectations at the end of the feedback-OFF period with those at the end of the feedback-ON period, centroid distance significantly increased by 7° for S1, 4° for S2, and 4° for S3. Figure 2 shows relative increases in localization errors when no feedback was provided.

Over the entire time that subjects used misaligned CAPs, none reported any problematic percepts. None of the subjects had difficulty using their systems or noticed any discrepancies between their visual percepts and their other senses. When asked to simultaneously view and hold an illuminated rod, subjects could detect changes in where they localized the light when different CAPs were set, but did not readily perceive any sensory discordance.

**Figure 3:**
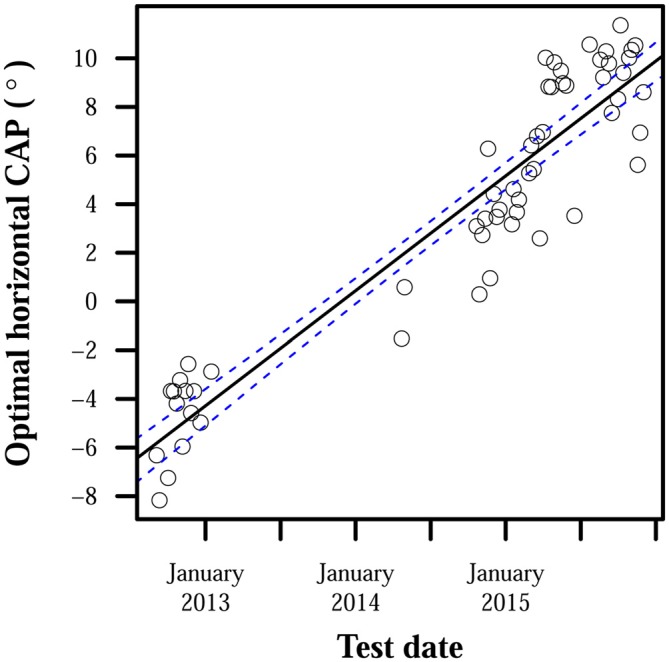
**Optimal horizontal camera alignment positions (CAPs) for S3 over time.** Positive ordinate values indicate rightward eccentricity in the camera’s visual field, and negative values indicate leftward eccentricity. Points each represent optimal horizontal CAP estimates averaged over all trials on 1 day. S3’s camera was intentionally misaligned during the period between January 2013 and April 2014, and collected data were therefore unsuitable for estimating S3’s optimal CAP. The line of best fit has a slope of 0.01°/day and intercept of −5.8°. The line’s 95% confidence bands are shown with dashed lines.

While little adaptation to misaligned CAPs was observed, CAPs required for proper alignment did fluctuate in all subjects. CAPs that provided optimal localization accuracy to subjects for a time eventually required adjustment to restore accuracy. MANOVA tests found significant effects of time: *p* < 10^−4^ for all subjects. Maximum differences between optimal CAPs for each subject were: 23° for S1, 29° for S2, and 21° for S3. Optimal CAP rates of change pooled across subjects had a median of 0.28°/day, mean of 0.39°/day, standard deviation of 0.36°/day, and maximum rate of 1.8°/day. Certain patterns did appear in how CAP estimates moved over time: optimal CAPs for S2 tended to move up and to the right over the observed period, and S3’s optimal CAPs moved very consistently to the right. Changes over time in S3’s optimal horizontal CAPs are highlighted in Figure 3. Other observed shifts were less predictable: S1 displayed a weak rightward trend and no apparent vertical trend over time, and S3’s vertical shifts only weakly trended downward.

**Figure 4:**
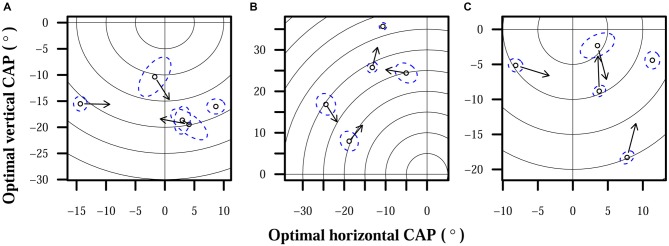
**Optimal CAPs for each subject.** Points each represent optimal CAP estimates averaged over all trials on 1 day. Each plot shows the field of view of the camera, focused on the region occupied by subject CAPs. The origin represents the center of the camera, and concentric rings are shown for each 5° of eccentricity. Dashed elliptical outlines mark 95% confidence regions for each CAP estimate. Arrows indicate chronological progression of optimal CAPs. **(A)** S1. **(B)** S2. **(C)** S3.

Figure 4 shows examples of subjects’ estimated optimal CAPs that differed significantly over time. For each subject, up to 4 points indicate the horizontal and vertical limits of optimal CAP positions and 1 point indicates the closest observation to the overall average optimal CAP. Arrows on the four first points in chronological order have arrows that point to the displayed CAP estimate that is next in chronological order.

## Discussion

Visual prostheses with extraocular cameras require calibration to optimize user hand-camera coordination. Camera input and/or processing can be changed to improve or degrade pointing accuracy. When users’ cameras were not properly configured, those in this study did not seem to fully appreciate the nature of the misalignments. Passive adaptation to misalignments, i.e., without specific instruction and coaching from someone such as a rehabilitation specialist, was possible, but only with very slow progress. Rates of adaptation seen here were about 4000 times slower than those for normally sighted subjects wearing prism glasses (Gibson, 1933). S2 did not show significant localization improvement while auditory feedback was enabled, in contrast to our two other subjects. S2 was less diligent in providing precise responses, which added more variability to localization data and may have accompanied paying less attention to auditory feedback. Both of these factors would make observing significant improvement less likely.

Without consistent auditory feedback on in-lab localization errors, pointing accuracy deteriorated for all of our subjects. Error magnitudes increased immediately after auditory feedback was removed for S1 and S3, and only gradually increased for S2. This difference could once again be explained by S2’s less diligent approach: if S2 was paying relatively little attention to the feedback, one would not expect removing the feedback to have as great an effect on responses. The gradual yet distinct increase in S2’s errors after feedback was removed, however, does suggest that the feedback worked to maintain the subject’s accuracy, if not improve it. For S1 and S3, the immediate increases in error magnitude may reflect the feedback acting as a reminder for the subjects to attend more carefully to how they respond, alongside providing information necessary for adaptation.

One might expect daily activities to provide corrective feedback on camera misalignments, such as reaching for a white mug against a dark background and missing. Unfortunately, subjects in this study did not appear to encounter or register enough of that information to improve or maintain pointing accuracy. It is possible that rehabilitation specialists familiar with visual prostheses and camera misalignments could teach users to detect and adjust to misalignments in their home environments. Further, a variation of the localization test used in this study that provides more precise feedback and allows users to make multiple attempts for one target could promote faster adaptation. The results of this study are restricted to contexts that do not involve specific coaching or devices designed to actively train users on correcting localization errors.

Lacking the ability to readily and independently adapt to misaligned percepts, the flexible nature of how prosthetic visual input is integrated into the perception of egocentric space is a point of concern. If users consistently required the same CAP to maintain hand-camera coordination, prosthesis systems would only need to be properly configured once. If a CAP initially set to maximize pointing accuracy becomes less suitable over time, however, and users cannot independently adapt to emergent misalignments, more frequent system calibrations will be required.

Further research will be necessary to better understand what causes perceived percept locations, and thus optimal CAPs, to change over time. Some of the variation seen here may stem purely from the alignment and measurement processes used in this study; however, the consistent trends displayed over time by S2 and S3 suggest that at least part of this variability was intrinsic to the subjects. If variability originating from the subject could be explained by something as simple as how the eye rests in the orbit, prosthesis-integrated eye tracking mechanisms may be able to adjust CAPs automatically. If more complicated problems are involved, such as changing alignments of visual and proprioceptive percepts, more involved rehabilitation training or device programming may be needed to maintain optimal hand-camera coordination.

Visual prostheses are starting to restore modest levels of vision to those without any other available treatments. As the technology improves, prostheses may one day provide enough visual information for users to passively adapt to misaligned percepts on their own. At that point, camera alignment could simply be a matter of preference. Until that time, however, prostheses that use extraocular cameras will need more configuration to optimize hand-camera coordination. Users who consider accurate coordination very important, more so than the subjects tested in this study, should have their cameras aligned on a regular basis to get the most benefit from their prostheses. Alternatively, such users could also seek training that may help them to actively detect and correct camera misalignments, if they are sufficiently motivated. None of our three subjects expressed any problems that would have necessitated such alignments or training.

## Disclaimer

Materials used for the described experiments were provided by Second Sight Medical Products, Inc. without charge. Johns Hopkins University received payment from Second Sight Medical Products, Inc. for participation in the Argus II Feasibility Study.

## Author Contributions

MPB: designed experiments, collected data, analyzed data, wrote manuscript. GD: designed experiments, edited manuscript.

## Funding

NIH T32 EY07143 to the Johns Hopkins Visual Neuroscience Training Program for MPB.

## Conflict of Interest Statement

The authors declare that the research was conducted in the absence of any commercial or financial relationships that could be construed as a potential conflict of interest.

## Footnotes

^1^Patients implanted with IRIS-1 (49 electrodes) 6 month after implantation (short version), n.d. (video file), Available online at: http://www.pixium-vision.com/en/clinical-trial/testimonials. [28 February 2016]

## Abbreviations

CAP: camera alignment position.
